# Inflammatory Immune Cytokine TNF-α Modulates Ezrin Protein Activation *via* FAK/RhoA Signaling Pathway in PMVECs Hyperpermeability

**DOI:** 10.3389/fphar.2021.676817

**Published:** 2021-05-12

**Authors:** Qun Zhou, Jianjun Jiang, Guanjun Chen, Cheng Qian, Gengyun Sun

**Affiliations:** ^1^ Department of Respiratory and Critical Care Medicine, The First Affiliated Hospital of Anhui Medical University, Hefei, China; ^2^ Department of Geriatric Respiratory Medicine, The First Affiliated Hospital of Anhui University of Chinese Medicine, Hefei, China; ^3^ The Center for Scientific Research of Anhui Medical University, Hefei, China

**Keywords:** acute respiratory distress syndrome, inflammatory immune response, endothelial permeability, Ezrin protein, molecular mechanism

## Abstract

**Background:** One of the important pathogenesis of acute respiratory distress syndrome (ARDS) is the dysfunction of pulmonary microvascular endothelial barrier induced by a hyperinflammatory immune response. However, the potential mechanisms of such an imbalance in pulmonary microvascular endothelial cells (PMVECs) are not yet understood.

**Purpose:** Explore the molecular mechanism of endothelial barrier dysfunction induced by inflammatory immune cytokines in ARDS, and find a therapeutic target for this syndrome.

**Methods:** Rat PMVECs were cultured to form a monolayer. Immunofluorescence, flow cytometry, and Western blotting were selected to detect the distribution and the expression level of phosphorylated Ezrin protein and Ezrin protein. Transendothelial electrical resistance (TER) and transendothelial fluxes of fluorescein isothiocyanate (FITC)-labeled bovine serum albumin (BSA) were utilized to measure the permeability of the cell monolayer. Ezrin short hairpin RNA (shRNA) and Ezrin 567-site threonine mutant (Ezrin^T567A^) were used to examine the role of Ezrin protein and phosphorylated Ezrin protein in endothelial response induced by tumor necrosis factor-alpha (TNF-α), respectively. The function of focal adhesion kinase (FAK) and Ras homolog gene family, member A (RhoA) signaling pathways were estimated by inhibitors and RhoA/FAK shRNA in TNF-α-stimulated rat PMVECs. The activation of FAK and RhoA was assessed by Western blotting or pull-down assay plus Western blotting.

**Results:** The TER was decreased after TNF-α treatment, while the Ezrin protein phosphorylation was increased in a time- and dose-dependent manner. The phosphorylated Ezrin protein was localized primarily at the cell periphery, resulting in filamentous actin (F-actin) rearrangement, followed by a significant decrease in TER and increase in fluxes of FITC-BSA. Moreover, FAK and RhoA signaling pathways were required in the phosphorylation of Ezrin protein, and the former positively regulated the latter.

**Conclusion:** The phosphorylated Ezrin protein was induced by TNF-α *via* the FAK/RhoA signaling pathway leading to endothelial hyperpermeability in PMVECs.

## Introduction

Acute respiratory distress syndrome (ARDS) is an acute respiratory failure caused by a variety of factors. It is a challenging clinical problem because of high mortality (40%) and limited treatment options (Bellani et al., 2016). The pathogenesis of ARDS is complex, involving different immune cells from innate and acquired immune systems to produce different inflammatory immune cytokines such as tumor necrosis factor-alpha (TNF-α) and ultimately leading to noncardiogenic pulmonary edema. Patients with ARDS afflicted with noncardiogenic pulmonary edema are caused by increased pulmonary microvascular permeability (Ranieri et al., 2012), in which the semipermeable barrier of pulmonary microvascular endothelial cells (PMVECs) plays a key role (Villar et al., 2019). The structure of PMVEC barrier is composed of tight junctions, adhesion junctions, and gap junctions between endothelial cells (ECs). Tight junctions bind to cytoskeletal actin through the cytoplasmic tail of transmembrane proteins such as occludin and claudin. Adhesion junctions and gap junctions bind to cytoskeletal actin *via* some proteins which can combine with the cytoplasmic tail of transmembrane proteins such as VE-cadherin and occludin. The adhesion between ECs and extracellular matrix is mediated by integrin and actin. These junctions are involved in maintaining the integrity of the endothelial barrier and related to the change in cytoskeletal actin (Bazzoni and Dejana, 2004; Naikawadi et al., 2012). Therefore, finding an intrinsic target for inhibiting filamentous actin (F-actin) recombination will be a new direction for studying the pathogenesis of ARDS.

Ezrin protein, a member of the ERM (Ezrin, radixin, and moesin) protein family, serves as a cross linker between F-actin and the plasma membrane. It exists in two convertible constitutions. A closed constitution in which the N and C termini are embraced in an intramolecular association or an open one that results from binding to phosphatidylinositol 4, 5-bisphosphate (PIP2), followed by phosphorylation of threonine 567 in the C-terminal F-actin-binding domain. The open constitution enables the Ezrin protein to link the plasmolemma to F-actin (Fievet et al., 2004). It can transmit different molecular signals on the surface of the membrane and execute various biological functions, such as cellular metabolism, adhesion, proliferation, and migration (Ben-Aissa et al., 2012). Despite the structural similarities of ERM and functional redundancy (Fievet et al., 2004), the roles of these three proteins in the endothelial barrier are different. For example, Adyshev et al. showed that radixin siRNA alone attenuates sphingosine-1 phosphate (S1P)-initiated human pulmonary artery EC barrier enhancement, and a partial attenuation can be obtained using Ezrin silencing, while moesin depletion exerts an opposite effect (Adyshev et al., 2011). Depletion of either moesin or ERM proteins attenuates thrombin-induced F-actin rearrangement and paracellular gap formation and decreases the transendothelial electrical resistance (TER), while radixin silence has an opposite effect on the endothelial barrier (Adyshev et al., 2013). Herein, an in-depth investigation of Ezrin protein is essential to study the regulatory mechanisms of PMVEC permeability. Mechanically, the signaling pathways are factually indispensable while studying Ezrin protein. In this study, Ras homolog gene family, member A (RhoA) and focal adhesion kinase (FAK) signaling pathways were considered in detail. Growing evidence reported that RhoA and FAK signaling pathway participates in endothelial hyperpermeability (Zhang et al., 2014; Alexopoulou et al., 2017; Zamorano et al., 2019). Moreover, FAK can regulate Ezrin protein (Bryant et al., 2014), and both could be related to Rho signaling pathways, especially RhoA (Huang et al., 2012).

To date, few studies have specifically addressed the involvement of Ezrin protein alone in the regulation of PMVEC monolayer permeability. We previously detected the expression level of phosphorylated Ezrin protein and found that it interacts with Rac1 signaling pathway to regulate PMVECs permeability (Tang et al., 2021). In this study, we intend to further synchronously observe the effects of the distribution and expression level of phosphorylated Ezrin protein on F-actin and endothelial permeability in TNF-α-stimulated PMVECs, and explore whether this process can be realized *via* RhoA and FAK signaling pathways, which, to our best knowledge, has not been reported. Encouragingly, it was found that after TNF-α treatment, the F-actin and the endothelial permeability were positively regulated by phosphorylated Ezrin protein in PMVECs, which was the downstream target of FAK/RhoA signaling events. This study will help to find the molecular targets for the treatment of ARDS in the downstream of complex inflammatory immune response.

## Methods and Materials

### Reagents

Recombinant rat TNF-α was purchased from PeproTech Inc (#400-14, Rochy Hill, NJ, United States). Ezrin and phospho-threonine^567^Ezrin (Thr567) polyclonal antibodies and high glucose Dulbecco’s Modified Eagle’s Medium (DMEM) were all purchased from Invitrogen (Ezrin, # PA5-82769, phospho-threonine^567^Ezrin, # PA5-37763, DMEM, SH30022.01, Carlsbad, CA, United States ). FAK and phospho-tyrosine^397^ FAK (Tyr397) antibodies were bought from Cell Signaling (FAK, # 3285, phospho-tyrosine^397^ FAK, # 3283, Danvers, MA, United States). C3 transferase was obtained from Cytoskeleton (# CT03, Denver, CO, United States). PF-573228, rhodamine-conjugated phalloidin, and fluorescein isothiocyanate phytohemagglutinin (FITC-BSI) were obtained from Sigma-Aldrich (PF-573228, PZ0117, rhodamine-conjugated phalloidin, P1951, FITC-BSI, L2895, St. Louis, MO, United States). FITC-labeled bovine serum albumin (FITC-BSA) and unlabeled BSA were procured from Beijing Solarbio Life Sciences (FITC-BSA, SF063, BSA, A8010, Beijing, China). Alexa Fluor 488-conjugated AffiniPure goat anti-rabbit IgG (H + L) was from Jackson Immumo Research Inc. (111–545–003, West Grove, PA, United States). We bought Horseradish peroxidase (HRP)-labeled antibody against rabbit IgG from Beijing Zhongshan Jinqiao Biotechnology Co., Ltd. (# ZB-2301, Beijing, China). The cell culture chambers of Transwell clear polyester membrane (12-well type, 12 mm diameter, 0.4 μm pore size) were gained from Costar (3460, Cambridge, MA, United States). Fetal bovine serum (FBS) was from Gibco (10099-141, CA, United States). Goat serum, Triton X-100, DNA-binding dye 2-(4-amidinophenyl)-6-indolecarbamidine dihydrochloride (DAPI), and antifade mounting medium were procured from Shanghai Absin Biotechnology Co., Ltd. (Goat serum, abs933, Triton X-100, abs9149, DAPI, abs47047616, antifade mounting medium, abs9234, Shanghai, China).

### Animals

The animal studies were ratified by the Institutional Animal Experimentation Ethics Committee of Anhui Medical University (approval no. LLSC20190752). All experiments conformed to the relevant regulations of animal protection. The adult male specific-pathogen-free Sprague–Dawley *Rattus norvegicus* (rats), aged 4.3 ± 0.2 weeks and weighing 115 ± 10 g, were provided by Anhui Medical University Animal Center, China (SCXK(Wan) 2017-001). The rats were outbred and maintained under a natural cycle of day and night with free access to water and food at ambient temperature (22–24°C).

### Cell Culture

After the animal study protocol was approved by the Institutional Animal Experimentation Ethics Committee of Anhui Medical University, the isolation and culture of primary rat PMVECs were performed as depicted previously (You et al., 2010). In simple terms, the fresh lung was aseptically taken down from the sacrificed male Sprague–Dawley rats. After the pleura was cut off, the outer edges of the remaining lung tissues without large blood vessels were collected. Then, we plated the tissues (1.5 mm^3^) into cell culture flasks containing 20% (vol/vol) FBS of DMEM under 5% (vol/vol) CO_2_ in an incubator (37°C). We dislodged the residue lung tissues after 60 h and changed the culture medium every 3 days. The cells were identified as PMVECs due to cobblestone morphology and positive FITC-BSI binding test as assessed by fluorescence microscope (Axio Observer 3, Zeiss, Oberkochen, Germany) (Supplementary Figure S4). The experimental information was gathered from cells in 3^rd^–5^th^ generations.

### Evaluation of Endothelial Monolayer Permeability

In order to construct a monolayer of cells *in vitro*, rat PMVECs (2 × 10^5^ cells/cm^2^) were seeded on gelatin-coated transwell polyester membranes, that is, rat PMVECs were seeded on transwell chamber in 12-well plates. The monolayer confluence of rat PMVECs was judged by TER and inverted microscope. Subsequently, the cells were cultured in a low-serum medium (DMEM supplemented with 1% FBS) for 24 h to allow synchronous growth up to a quiescent phase, followed by incubation with normal culture medium (DMEM supplemented with 20% FBS) with or without stimuli. The change in the endothelial permeability was detected by both TER and trans-endothelial flux of FITC-BSA. TER was examined using an epithelial volt–ohm meter and STX-2 electrodes (EVOM; World Precision Instruments, Sarasota, FL, United States) in accordance with the operating instructions of the instrument. To measure the trans-endothelial flux of FITC-BSA across the endothelial monolayer of cultured rat PMVECs, after stimulation, fresh phenol red-free DMEM containing FITC-BSA (0.5 mg/ml) was added on the top chamber and an equimolar amount of unlabeled BSA mixed with phenol red-free DMEM was put into the bottom chamber to maintain isotonic condition, the chamber was incubated in an incubator (1 h, 37°C). The samples were collected from the top and bottom chamber and analyzed on a multimode plate reader (EnSpire^®^, PE, United States) at excitation 493 nm and emission 550 nm wavelengths. We calculated the flux of FITC-BSA *via* the rate of fluorescence intensity in the bottom chamber (1 h) and the top chamber (0 h) (Yang et al., 2010). The data were exhibited as a ratio of change value to the control group.

### Immunofluorescence

5 × 10^4^ rat PMVECs were incubated on the coverslips (2 cm^2^). The cells were first observed under an inverted microscope, and then relevant reagents were added to carry out the experiment unless the cells fused into a monolayer. After the drug stimulated, the coverslips were rinse by 1x phosphate buffer saline (PBS) (pH 7.4). The cells were fixed with 4% (wt/vol) paraformaldehyde (pH 7.4) for 15 min, blocked with blocking buffer (1 × PBS, 5% (vol/vol) goat serum, 0.3% (vol/vol) Triton X-100) for 1 h, and mixed with Ezrin or phospho-threonine^567^Ezrin polyclonal anti-rabbit antibodies overnight at 4°C, followed by incubation with the fluorescent secondary antibody (Alexa Fluor 488–conjugated AffiniPure goat anti-rabbit IgG (H+L)) for 2 h at room temperature. Fixed cells were mixed with rhodamine-conjugated phalloidin to stain for F-actin (1 h, 37°C) and subsequently with DAPI to stain the nucleus for 30 min at room temperature. The coverslips were mounted on glass slides with antifade mounting medium and examined using a confocal laser scanning microscope (LSM880 + airyscan, Ziess, Oberkochen, Germany). In addition, some of the incubations were performed in the dark to avoid loss of activity of the fluorochromes. All of the images were captured *via* 40x oil lens (numerical aperture of 1.3) at room temperature. The images were gained *via* the manufacturer’s software (Zen software, Oberkochen, Germany), and the acquired images were not post-processed.

### Flow Cytometry

After confluence, rat PMVECs were detached with 0.25% (wt/vol) trypsin, fixed with 4% (wt/vol) paraformaldehyde (pH 7.4) for 15 min, punched with 0.3% (vol/vol) Triton X-100, incubated with Ezrin or phospho-threonine^567^Ezrin antibody (2 h, 37°C), and then mixed with fluorescent second antibody (1 h, 37°C). After washing, the cells were resuspended in rhodamine-conjugated phalloidin (1 h, 37°C), and analyzed by CytoFLEX flow cytometry (Beckman, Brea, United States) and displayed as one-parameter histograms. The quantitative analysis of fluorescence data was carried out by CytoFLEX software, and the protein fluorescence were expressed by the mean fluorescence intensity (MFI) of the experimental samples.

### Western Blotting

Whole protein was extracted from cell samples in the following way: when monolayer cells were fused, 1 × PBS was used to rinse the cells three times, and then 1% (vol/vol) phenylmethanesulfonyl fluoride (PMSF) of RIPA lysis buffer (50 mM Tris (pH 7.4), 150 mM NaCl, 1% sodium deoxycholate, 1% Triton X-100, and 0.1% SDS) was added. After 30 min on ice, the lysate containing cells was collected and centrifuged (10 min, 4°C, 12,000 rpm), and the supernatant was gathered. The protein concentration was detected using the Bradford Protein Assay Kit (Beyotime, Shanghai, China). An equivalent of 20 μg protein was loaded per well and fractionated on 10–12% (vol/vol) sodium dodecyl sulfate-polyacrylamide gel electrophoresis (SDS-PAGE) and devolved to a polyvinylidene difluoride membrane by using the wet Western blotting transfer mode. Next, the membrane was blocked with 5% (wt/vol) nonfat milk of TBST (1 × TBS, 0.1% (vol/vol) Tween 20, and pH 7.4) at room temperature (2 h), incubated with the primary antibodies overnight (4°C). Subsequently, the membrane was put into HRP-conjugated secondary antibodies for 1 h at room temperature and developed using an enhanced chemiluminescence detection system (AL600RGB, GE Healthcare Life Sciences, Chalfont, United Kingdom). The blots were quantitatively analyzed by NIH-Image J 1.45 (National Institutes of Health, Bethesda, MD, United States).

### RhoA Activation Assay

RhoA activity was assessed *via* a pull-down assay using the RhoA Activation Assay Kit (Cytoskeleton Inc. Denver, CO, United States), according to the manufacturer’s guidance. Briefly, after stimulation, PMVECs were lysed with cell lysis buffer (50 mM Tris pH 7.5, 10 mM MgCl_2_, 0.5 M NaCl, and 2% (vol/vol) Igepal), and GTP-bound RhoA (RhoA-GTP) was captured when the lysates were incubated with Rhotekin RBD beads (1 h, 4°C). The beads were washed four times with wash buffer (40 mM NaCl, 30 mM MgCl_2_, and 25 mM Tris pH 7.5) and heated to 95°C for 2 min with 20 µl of 2x Laemmli sample buffer (125 mM Tris pH 6.8, 20% (vol/vol) glycerol, 4% (wt/vol) SDS, 0.005% (wt/vol) bromophenol blue, 5% (vol/vol) beta-mercaptoethanol), and then loaded on a 10% (vol/vol) SDS-PAGE. The expression level of RhoA-GTP was caught by Western blotting using a monoclonal antibody against RhoA. In cell lysates, the total amount of RhoA was used as an endogenous control.

### shRNA Silencing

The lentiviruses were packaged using a four-plasmid system as proposed before (Tiscornia et al., 2006). The lentivirus particles (GenePharma Co., Ltd., Shanghai, China), which express short hairpin RNA (shRNA) or a negative control shRNA (targeting Ezrin shRNA sequence, GGA​TCA​ACT​ATT​TCG​AGA​TCA; targeting RhoA shRNA sequence, AAG​GAT​CTT​CGG​AAT​GAT​GAG; targeting FAK shRNA sequence, AAG​CTG​CTG​AAC​TCC​GAC​TTG; negative control sequence, GTT​CTC​CGA​ACG​TGT​CAC​GT), were used to infect PMVECs that were seeded in a 6-well plate (1 × 10^6^ cells/well). When the cells reached 40–60% confluence, the medium was replaced with 20% FBS of DMEM, lentiviruses, and 5 μg/ml polybrene for 24 h. Then, the supernatant with lentivirus particles was replaced by the normal culture medium containing 20% FBS for 48 h. The effect of lentivirus transfection was observed under a fluorescence microscope (Supplementary Figure S5) and that of gene knockdown was examined by Western blotting.

### Construction of Ezrin Threonine 567-Site Mutant

Using Ezr NM-019357-WT (Ezrin^WT^) as template, Ezrin 567-site threonine was mutated to alanine (Ezrin^T567A^). After constructing the plasmid and packaging the lentivirus (GenePharma Co., Ltd., Shanghai, China), the lentivirus was used to infect rat PMVECs. Rat PMVECs were spread in a 6-well plate (1 × 10^6^ cells/well), when the cells reached 40–60% confluence, they were incubated with 20% FBS, lentiviruses, and 5 μg/ml polybrene for 24 h, then with normal medium for 48 h. The transfection efficiency was detected by fluorescence microscope, and the knockdown effect was observed by Western blotting.

### Cell Viability Assay

Cell Counting Kit-8 (CCK8; BB-4202, BestBio, Shanghai, China) was used for detecting the viability of PMVECs. Firstly, PMVECs were seeded into microplates (96-well, 10^4^ cells/well), cultured overnight. Then, different concentration of TNF-α (0, 0.2, 2, 20, 200 ng/ml) was added to each well for 2 h. After that, 10 *µ*l of CCK8 was incubated with PMVECs for another 3 h. The optical density (OD) was detected at 450 nm *via* a multimode plate reader (EnSpire^®^, PE, United States).

### Statistical Analysis

Data was exhibited as mean ± SD. Statistical comparisons were done using one-way analysis of variance (ANOVA), pairwise comparisons using Student–Newman–Keuls (SNK) test, and least significant difference (LSD) test *via* SPSS 17.0 software. *p* < 0.05 indicated statistical significance.

## Results

### Phosphorylated Ezrin Protein Was Increased and TER Was Decreased in TNF-α-Stimulated PMVECs

To illuminate the effect of TNF-α on the Ezrin protein phosphorylation at its critical threonine residue, phospho-Ezrin (Thr567) polyclonal antibody was utilized. Western blotting result showed that phosphorylated Ezrin protein was enhanced in a time-dependent manner induced by 20 ng/ml of TNF-α, which was elevated after 0.5 h, and reached a peak by 2 h and was maintained for at least 12 h, but without detectable change in the total Ezrin protein expression level (Supplementary Figures S1A,C). A dose-response result indicated that a significant increase in TNF-α-induced Ezrin protein phosphorylation was detected in the 20 ng/ml group as compared to the 0.2 ng/ml and 2 ng/ml groups, respectively (Supplementary Figures S1B,D). In the 200 ng/ml group, a marked increase was observed in the expression level of phosphorylated Ezrin protein. The expression level of Ezrin protein remained unchanged.

To observe the role of TNF-α in PMVECs permeability, the TER method was adopted. The result of analysis displayed that when challenged with TNF-α, the confluent PMVECs led to a decrease in TER in a period of 0–12 h and dose-dependent (0.2–200 ng/ml) manner (Supplementary Figure S2). In addition, our data statistically declared an obviously difference in TER between the control group and the 2 h group. However, no significant difference was detected among 2, 3, 6, and 12 h groups (*p* > 0.05).

Taken together, TNF-α induced the increase of phosphorylated Ezrin protein and PMVECs barrier damage in a time- and dose-dependent manner. In view of the data, it seems that 2 h and 200 ng/ml should be selected as the best choice (Supplementary Figures S1, S2). However, the cell viability in 200 ng/ml group was decreased as assessed by the CCK-8 assay (Supplementary Figure S3). As such, we believed that it was due to the cytotoxicity in the 200 ng/ml group, which might lead to cell death and endothelial monolayer damage. Based on these and previous results (Koss et al., 2006; Fang et al., 2018), we chose 20 ng/ml as the concentration of TNF-α and a duration of 2 h to conduct the follow-up experiments.

### Phosphorylated Ezrin Protein and Endothelial Hyperpermeability Was Inhibited by Ezrin shRNA in TNF-α-Stimulated PMVECs

**FIGURE 1:**
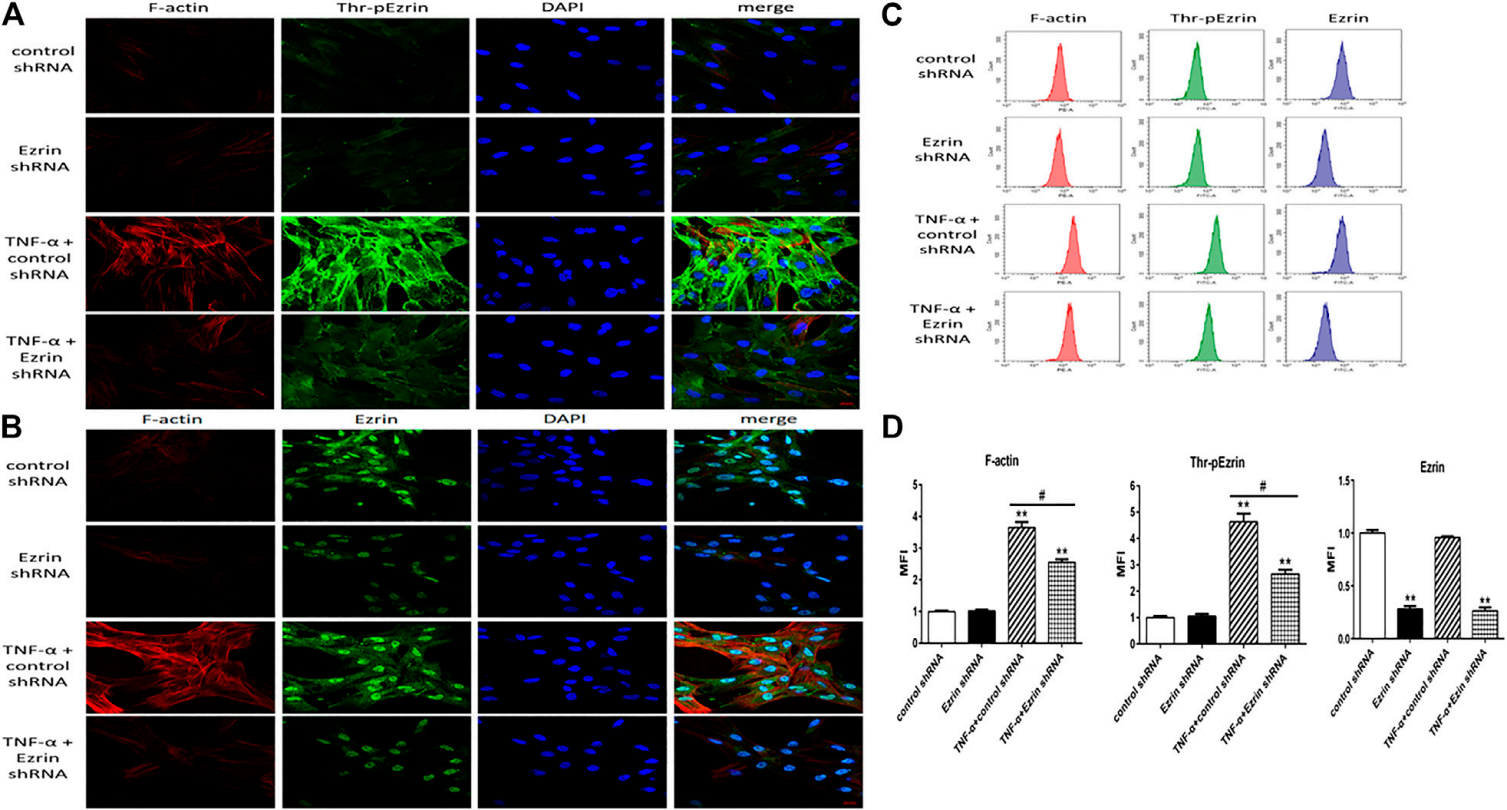
Effect of Ezrin shRNA on the rearrangement of F-actin and the distribution of threonine phosphorylated Ezrin/Ezrin protein (Thr-pEzrin/Ezrin). **(A)** For each panel, the images from left to right showed rhodamine fluorescence (red), Alexa Fluor 488 fluorescence (green), cell nuclei stained with DAPI (blue), and overlays of the three images. In control shRNA cells, few stress fibers were observed in the cytoplasm and phosphorylated Ezrin protein was primarily located in the cytoplasm. Ezrin shRNA showed a phenomenon similar to control one. Control shRNA cells exposure to 20 ng/ml TNF-α for 2 h induced F-actin recombination and gather of phosphorylated Ezrin protein to cell periphery. Downregulation of Ezrin protein weakened the F-actin band and induced a decrease of phosphorylated Ezrin protein locating to cell periphery. **(B)** The distribution of Ezrin protein was not altered by TNF-α while F-actin had a similar trend to **(A)**. Scale bar, 20 μm. **(C)** The fluorescence intensity of F-actin, phosphorylated Ezrin protein and Ezrin protein was detected *via* flow cytometry. **(D)** The mean fluorescence intensity (MFI) from flow cytometry. For each condition, the experiment was repeated three times, all of which had similar observations. Data was presented as mean ± SD. *n* = 3, **p < 0.01 vs. control group. ^#^ *p* < 0.05 between TNF-α + control shRNA and TNF-α + Ezrin shRNA groups.

To clarify the role of Ezrin protein in the PMVECs endothelial barrier stimulated by TNF-α, Ezrin shRNA was used. Immunofluorescence was considered to observe the distribution of F-actin, phosphorylated Ezrin protein, and Ezrin protein; flow cytometry was used to quantitatively analyze fluorescence intensity of them. Western blotting assay was applied to examine the expression levels of phosphorylated Ezrin protein and Ezrin. The TER and fluxes of FITC-BSA were adopted to further assess TNF-α-induced endothelial permeability. Colabeling F-actin (rhodamine-conjugated phalloidin, red) and phosphorylated Ezrin protein or Ezrin protein (Alexa Fluor 488 fluorescent secondary antibody, green) were used to detect the localization of them in TNF-α-stimulated PMVECs. The results of double immunofluorescence showed that before TNF-α treatment, the phosphorylated Ezrin protein could be detected both in the cytoplasm and at the cell border, and the F-actin was distributed thinly on the cell periphery. Moreover, the phosphorylated Ezrin protein was similar to each other while treating with the control shRNA and the Ezrin shRNA, respectively, and so was the F-actin. Of note, after TNF-α treatment in the control shRNA-treated PMVECs, the phosphorylated Ezrin protein was localized primarily on the cell periphery, together with F-actin reorganization (parallel bunchy accumulation and thickening). Conversely, Ezrin shRNA plus TNF-α prevented these change in PMVECs (Figure 1A). In addition, similar change occurred with respect to the distribution of F-actin in the four cases but Ezrin protein was not affected by TNF-α (Figure 1B). The result of MFI from flow cytometry showed that higher levels of both phosphorylated Ezrin protein and F-actin had been obtained in TNF-α-induced group compared to the control one. Importantly, after Ezrin shRNA was used, the MFI of them were partially reversed. Although the MFI of Ezrin protein was decreased significantly after Ezrin shRNA treatment, it was not affected by TNF-α (Figures 1C,D).

**FIGURE 2:**
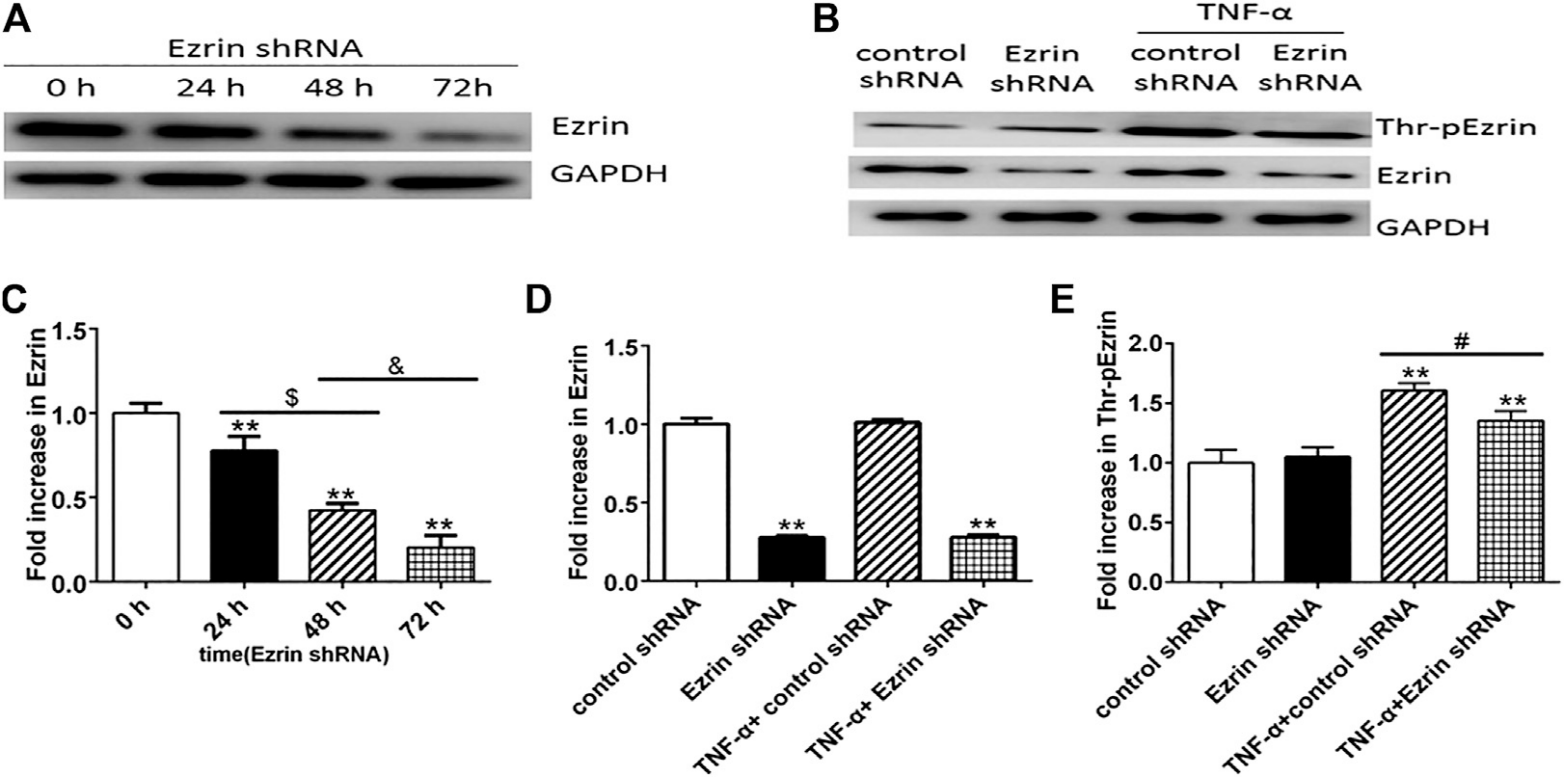
Effect of Ezrin shRNA on the expression level of Thr-pEzrin in TNF-α-induced PMVECs. **(A)** Ezrin shRNA downregulated the expression level of Ezrin protein. **(B)** Ezrin protein was not sensitive to TNF-α (20 ng/ml, 2 h) stimulation. Ezrin shRNA alleviate the increase of Thr-pEzrin induced by TNF-α. **(C–E)** Relative expression levels from the Western blotting. Data were presented as mean ± SD. *n* = 3, **p < 0.01 vs. control group. ^$^ *p* < 0.05 between 24 h and 48 h groups. ^&^ *p* < 0.05 between 48 h and 72 h groups, ^#^ *p* < 0.05 between TNF-α + control shRNA and TNF-α + Ezrin shRNA groups.

**FIGURE 3:**
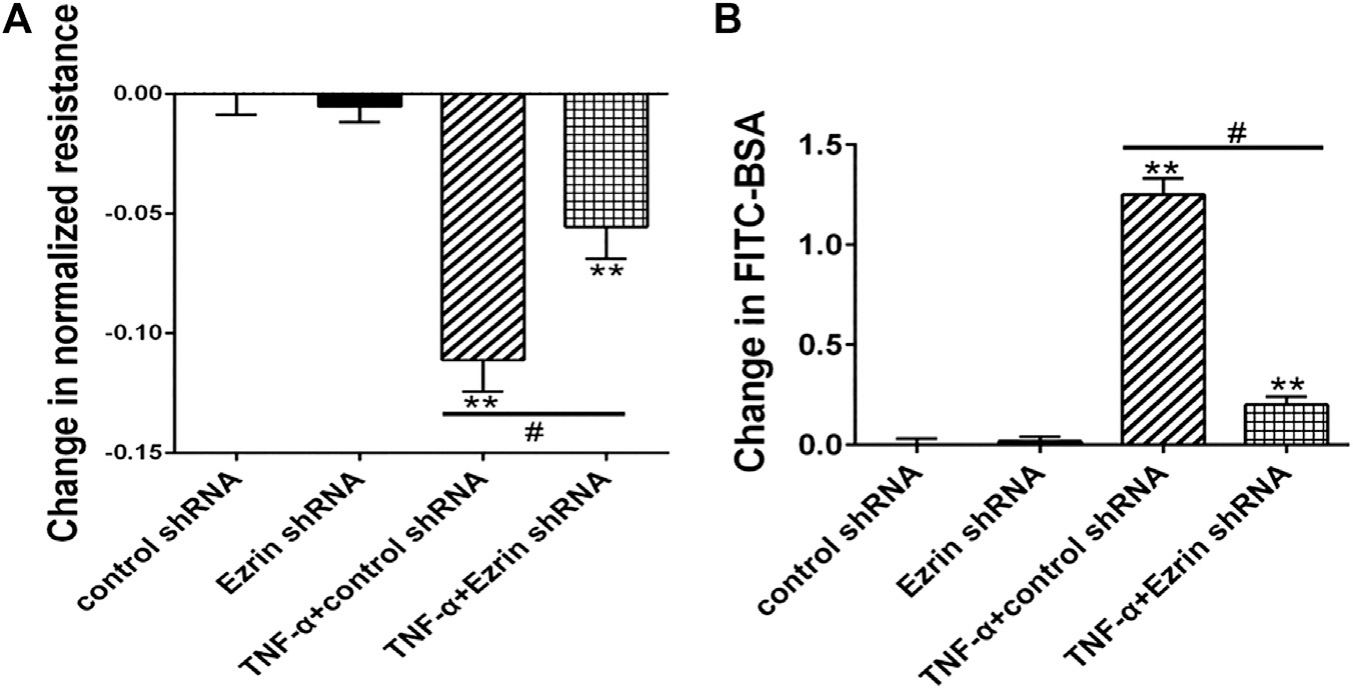
Effect of Ezrin shRNA on endothelial hyperpermeability in TNF-α-induced PMVECs. **(A)** Effect on TER of PMVECs monolayer. **(B)** Effect on fluxes of FITC-BSA across PMVECs monolayer. Each bar represented mean ± SD of three independent trials; ^**^ *p* < 0.01 vs. control group. ^#^ *p* < 0.05 between the TNF-α + control shRNA group and the TNF-α + Ezrin shRNA group.

Western blotting result revealed that Ezrin shRNA significantly downregulated the expression level of Ezrin protein (Figures 2A,C), but the expression level of Ezrin protein was not affected by TNF-α (Figures 2B,D). Notably, Ezrin shRNA could downregulate TNF-α-treated phosphorylated Ezrin protein expression level (Figures 2B,E). In addition, in the resting state, the expression level of phosphorylated Ezrin protein in the control group was similar to that in the Ezrin shRNA group (Figures 2B,E). This may be that the amount of phosphorylated Ezrin protein was very small in the resting state, and slightly affected by Ezrin shRNA, which was consistent with the resting state seen in Figure 1. Both the change in TER and the fluxes of FITC-BSA could be inhibited by Ezrin shRNA in TNF-α-stimulated PMVECs (Figure 3). Based on these results, we could conclude that Ezrin shRNA protected endothelial barrier through downregulating the distribution and the expression level of phosphorylated Ezrin protein in TNF-α-stimulated PMVECs.

### Phosphorylated Ezrin Protein Promoted Endothelial Hyperpermeability in TNF-α-Stimulated PMVECs

**FIGURE 4:**
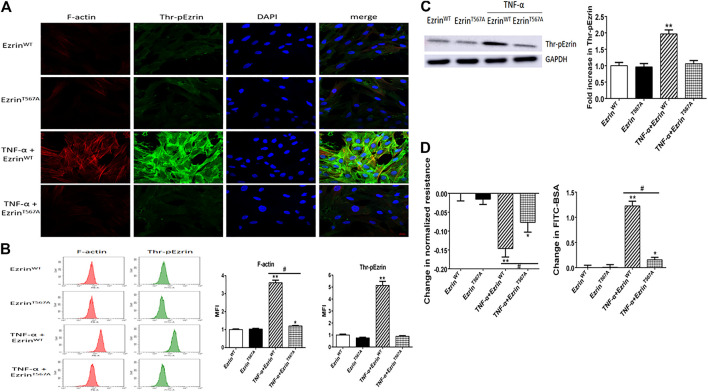
Effect of phosphorylation mutant of Ezrin protein (Ezrin^T567A^) on endothelial responses in TNF-α-induced PMVECs. **(A)** Ezrin^T567A^ can reverse the localization of Thr-pEzrin and reduce the recombination of F-actin induced by TNF-α. Scale bar, 20 μm. **(B)** The MFI of F-actin, Thr-pEzrin was calculated *via* flow cytometry. **(C)** Effect of Ezrin^T567A^ on the Thr-pEzrin expression level induced by TNF-α. **(D)** Ezrin^T567A^ relieved the decline of TER and increased the fluxes of FITC-BSA induced by TNF-α. For each condition, the experiment was repeated three times, all of which had similar observations. Data were presented as mean ± SD. *n* = 3, ^*^ *p* < 0.05 vs. control group. **p < 0.01 vs. control group. ^#^ *p* < 0.05 between TNF-α + Ezrin^WT^ and TNF-α + Ezrin^T567A^ groups.

To further evaluate the role of threonine-phosphorylated Ezrin protein in endothelial response to TNF-α, Ezrin threonine 567-site mutant (Ezrin^T567A^) was used. Immunofluorescence result showed that after TNF-α treatment, phosphorylated Ezrin protein gathered to the cell boundary in the Ezrin^WT^ group, but there was no obvious change in the Ezrin^T567A^ group, indicating that phosphorylated Ezrin protein achieved silencing effect. Ezrin^T567A^ could alleviate the aggregation of F-actin (Figure 4A). The result of flow cytometry analysis showed that Ezrin^T567A^ could block Ezrin protein phosphorylation and reduce the fluorescence intensity of F-actin in TNF-α-induced PMVECs (Figure 4B). Western blotting result revealed that Ezrin^T567A^ could reverse the expression level of phosphorylated Ezrin protein stimulated by TNF-α to the baseline level (Figure 4C). Ezrin^T567A^ could partially reverse the decrease of TER and the increase of FITC-BSA induced by TNF-α, which means Ezrin^T567A^ could protect the endothelial barrier (Figure 4D). These results indicated that phosphorylated Ezrin protein positively regulated endothelial hyperpermeability in PMVECs treated by TNF-α.

### Phosphorylated Ezrin Protein Was Increased *via* RhoA or FAK Signaling Pathway in TNF-α-Stimulated PMVECs

To determine the signal mechanisms of Ezrin protein phosphorylation with TNF-α treatment, the roles of RhoA and FAK signaling pathways were detected *via* inhibitors and signaling pathway knockdown, respectively.

**FIGURE 5:**
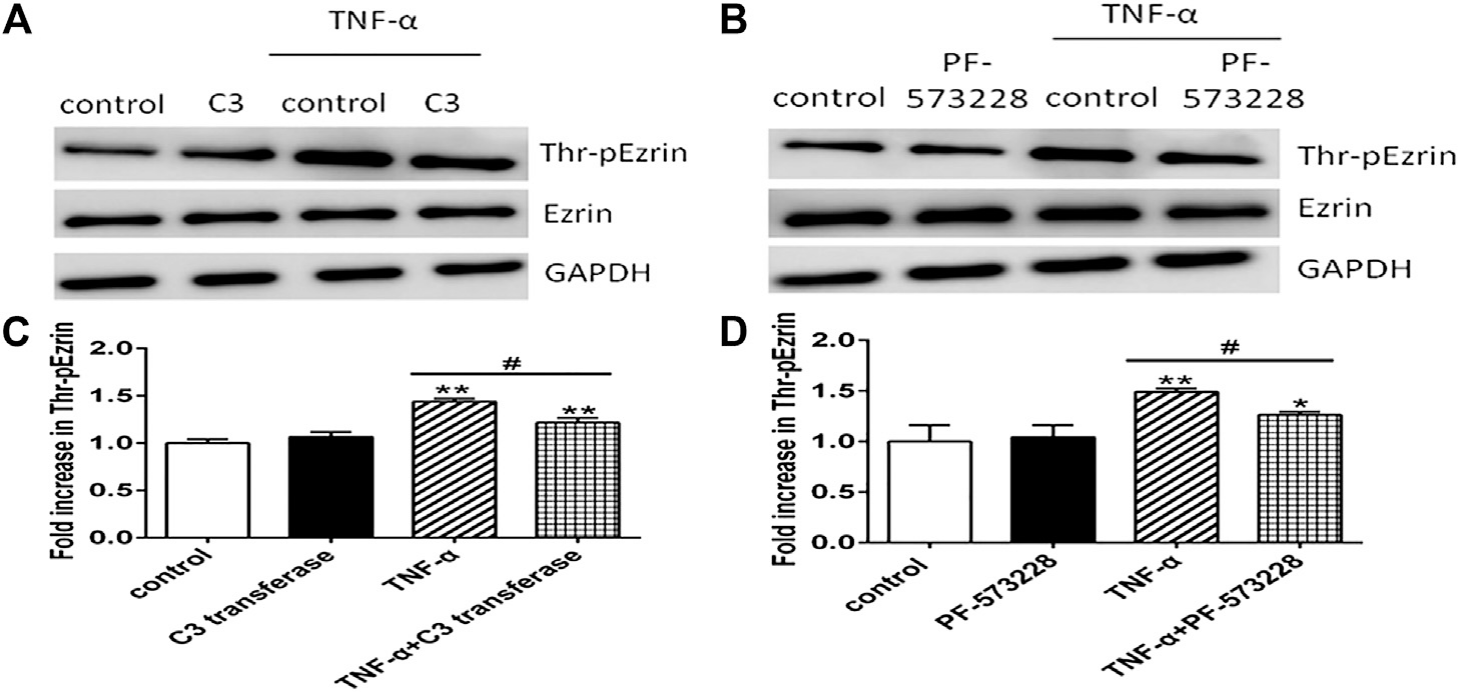
Effect of RhoA or FAK inhibitor on the expression level of Thr-pEzrin in TNF-α-induced PMVECs. **(A)** RhoA inhibitor prevented TNF-α-induced Ezrin phosphorylation. PMVECs were pretreated with either vehicle or 2 μg/ml C3 transferase for 6 h before they were treated with 20 ng/ml TNF-α for 2 h **(B)** FAK inhibitor alleviated TNF-α–induced Ezrin phosphorylation. PMVECs were pretreated with either vehicle or 1 μM PF-573228 for 0.5 h before they were treated with 20 ng/ml TNF-α for 2 h **(C,D)** The according expression level of phosphorylated Ezrin protein from the Western blotting. Data were presented as mean ± SD. *n* = 3, ^*^ *p* < 0.05, and **p < 0.01 compared to the control group. ^#^ *p* < 0.05 between the TNF-α group and the TNF-α + inhibitor group.

**FIGURE 6:**
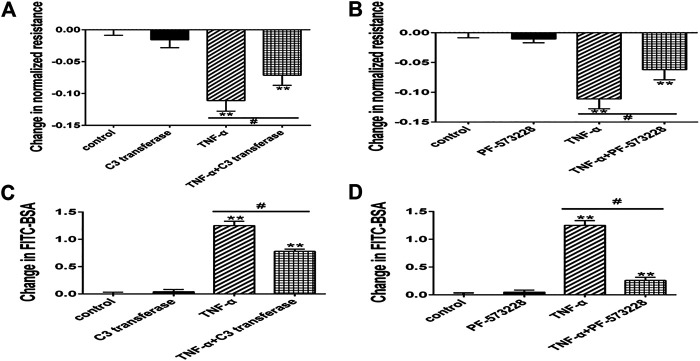
Effect of RhoA or FAK inhibitor on endothelial hyperpermeability in TNF-α-induced PMVECs. **(A,B)** RhoA or FAK inhibitor protected the decrease of TER treated with TNF-α. **(C, D)** RhoA or FAK inhibitor reduced the fluxes of FITC-BSA. Data were presented as mean ± SD. *n* = 3, **p < 0.01 compared to the control group. ^#^ *p* < 0.05 between the TNF-α group and the TNF-α + inhibitor group.

Firstly, RhoA inhibitor C3 transferase (Sun et al., 2006) and FAK inhibitor PF-573228 (Che et al., 2020) were used. We applied Western blotting to exhibit the expression levels of phosphorylated Ezrin protein and Ezrin protein, TER, and fluxes of FITC-BSA to observe TNF-α-induced endothelial permeability. C3 transferase could reduce Ezrin protein phosphorylation in TNF-α-stimulated PMVECs. A similar result was obtained when another inhibitor PF-573228 was used. Ezrin protein had no change in each group (Figure 5). Both inhibitors could conspicuously block the decrease in TER and the increase in FITC-BSA (Figure 6). The data declared that RhoA or FAK signaling pathway might enhance Ezrin protein phosphorylation in TNF-α-stimulated PMVECs.

**FIGURE 7:**
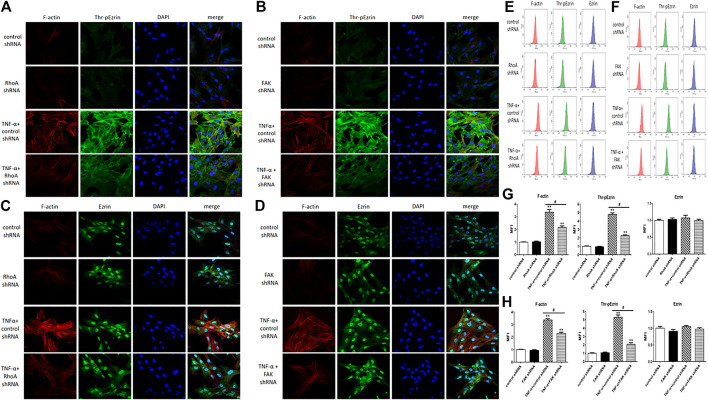
Effect of RhoA shRNA or FAK shRNA on the rearrangement of F-actin and the distribution of Thr-pEzrin/Ezrin. **(A,B)** The TNF-α responses were prevented remarkably *via* RhoA shRNA and FAK shRNA treatment, respectively **(C,D)** There was no change in Ezrin protein under the same stimulation. Scale bar, 20 μm. **(E,F)** The fluorescence intensity of F-actin, Thr-pEzrin/Ezrin was detected *via* flow cytometry. **(G,H)** MFI from flow cytometry. All images were representative of three independent experiments with similar observations. Data were presented as mean ± SD. *n* = 3, **p < 0.01 vs. control group. ^#^ *p* < 0.05 between TNF-α + control shRNA and TNF-α + RhoA/FAK shRNA groups.

**FIGURE 8:**
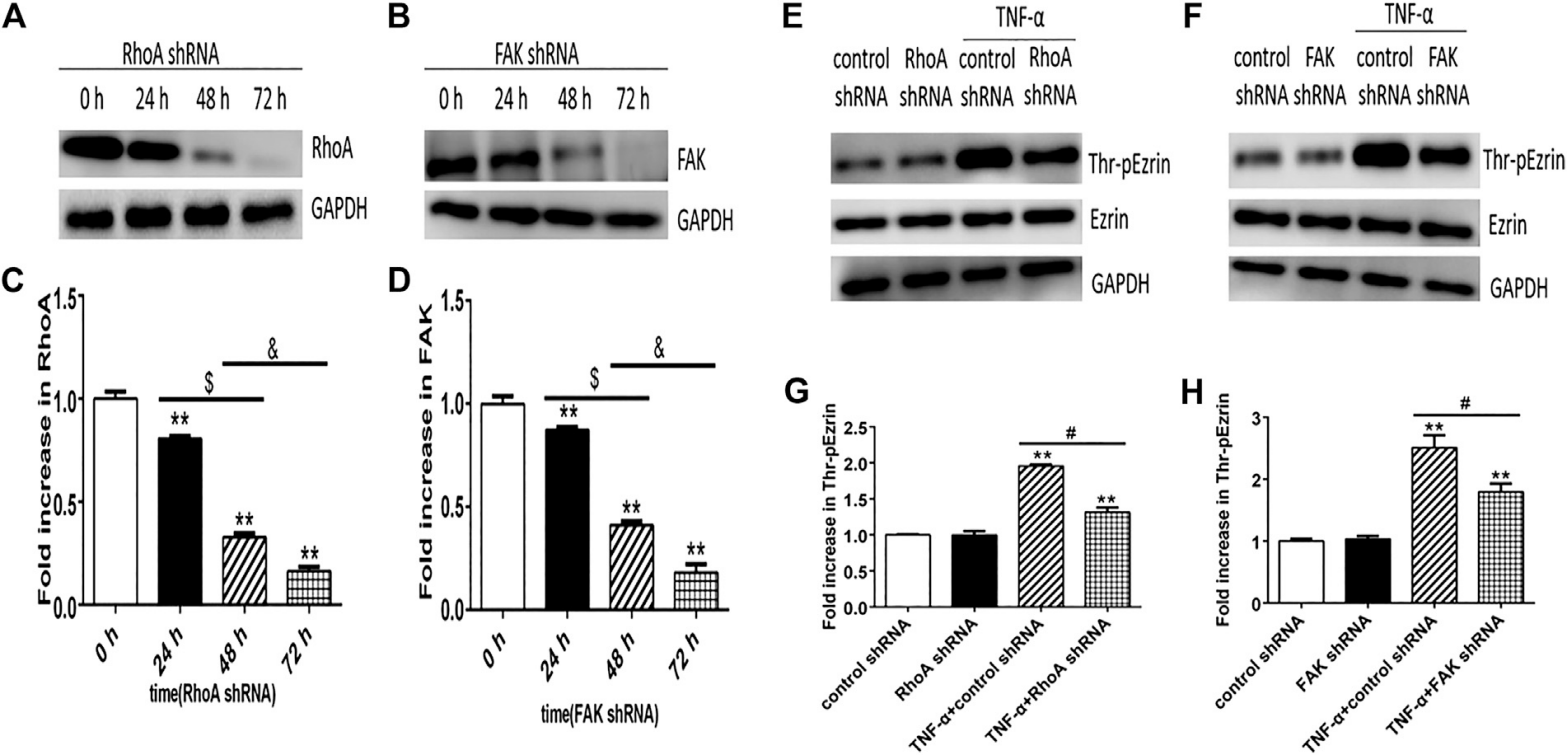
Effect of RhoA shRNA or FAK shRNA on the expression level of Thr-pEzrin in TNF-α-induced PMVECs. **(A)** RhoA shRNA downregulated the expression level of RhoA. **(B)** After FAK shRNA treatment, the expression level of FAK decreased. **(C,D)** The according expression levels from the Western blotting **(E,F)** Pretreatment with RhoA shRNA prevented TNF-α–induced Ezrin phosphorylation and so can FAK shRNA. **(G,H)** The according expression levels from the Western blotting. Data were presented as the mean ± SD. *n* = 3, **p < 0.01 compared to the control group. ^$^ *p* < 0.05 between 24 h and 48 h groups. ^&^ *p* < 0.05 between 48 h and 72 h groups, ^#^ *p* < 0.05 between TNF-α + control shRNA and TNF-α + RhoA/FAK shRNA groups.

**FIGURE 9:**
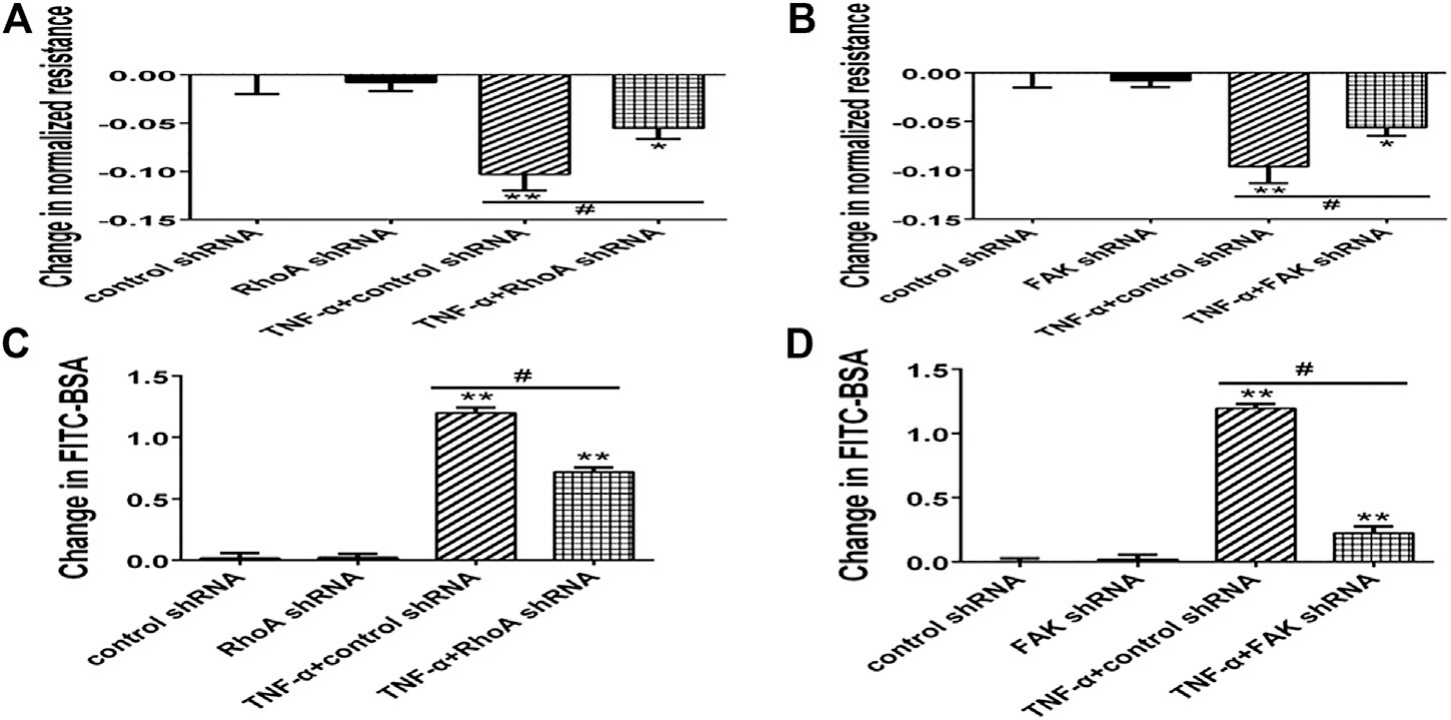
Effect of RhoA shRNA or FAK shRNA on endothelial hyperpermeability in TNF-α-induced PMVECs. **(A,B)** RhoA or FAK shRNA protected the decrease of TER treated with TNF-α. **(C,D)** RhoA or FAK shRNA decreased the fluxes of FITC-BSA. Data were presented as mean ± SD. *n* = 3, ^*^ *p* < 0.05 compared to the control group. **p < 0.01 compared to the control group. ^#^ *p* < 0.05 between TNF-α + control shRNA and TNF-α + RhoA/FAK shRNA groups.

Next, we selected RhoA shRNA and FAK shRNA to verify the roles of RhoA and FAK, respectively. Immunofluorescence result showed that TNF-α induced the F-actin reorganization and the location of phosphorylated Ezrin protein to the cell periphery. Treatment with RhoA shRNA or FAK shRNA prevented the TNF-α response obviously, respectively (Figures 7A,B). The distribution of Ezrin protein did not change in all groups (Figures 7C,D). Flow cytometry result also showed that RhoA shRNA or FAK shRNA could inhibit the increase of both F-actin and phosphorylated Ezrin protein induced by TNF-α but had no effect on Ezrin protein (Figures 7E–H). Afterward, the result of Western blotting analysis indicated that RhoA shRNA or FAK shRNA could downregulate the expression level of RhoA or FAK (Figures 8A–D). After RhoA shRNA or FAK shRNA treatment, the expression level of phosphorylated Ezrin protein was decreased significantly stimulated by TNF-α while Ezrin protein remained the same expression level (Figures 8E–H). The experimental results on the endothelial permeability also showed that RhoA shRNA or FAK shRNA could alleviate TNF-α-induced change in TER and fluxes of FITC-BSA (Figure 9). Based on these observations, we concluded that RhoA or FAK signaling pathway could promote Ezrin protein phosphorylation, resulting into the endothelial hyperpermeability in TNF-α-induced PMVECs.

### FAK Positively Regulated RhoA Signaling Pathway in TNF-α-Stimulated PMVECs

**FIGURE 10:**
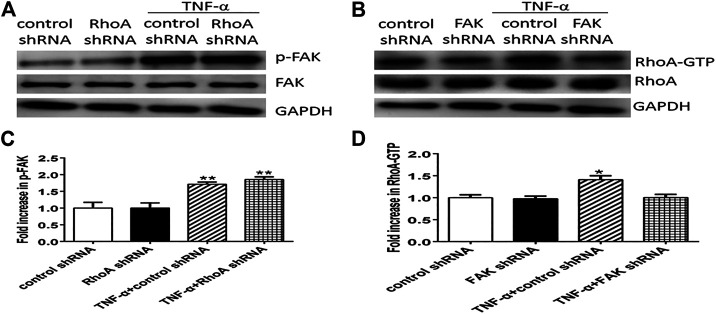
The FAK signaling pathway can upregulate the activity of RhoA in TNF-α-induced PMVECs. **(A)** RhoA shRNA cannot block FAK phosphorylation (p-FAK) induced by TNF-α. **(B)** FAK shRNA can inhibit RhoA activity (RhoA-GTP). **(C,D)** Relative expression levels from the Western blotting. Data were presented as mean ± SD. *n* = 3, ^*^ *p* < 0.05, **p < 0.01 compared to the control group.

To further clarify whether RhoA signaling pathway is the upstream of FAK in Ezrin protein phosphorylation, or vice versa, we considered the effect of RhoA shRNA (FAK shRNA) on the expression level of FAK (RhoA) by Western blotting. Firstly, we found that the expression levels of both RhoA-GTP and phosphorylated FAK were enhanced in TNF-α-stimulated PMVECs while RhoA and FAK had no change in each group. Then, we detected the effect of RhoA shRNA on FAK, no change of phosphorylated FAK occurred in the two cases of TNF-α alone and RhoA shRNA plus TNF-α. In other word, the pretreatment with RhoA shRNA did not exert an effect on FAK (Figures 10A,C). Next, we examined the effect of FAK shRNA on RhoA. It was found that RhoA-GTP was increased remarkably by TNF-α, while the combination of FAK shRNA and TNF-α evidently restored the increased expression level of RhoA-GTP almost to the original level (Figures 10B,D). Thus, it can be deduced that pretreatment with FAK shRNA restrained the activation of RhoA. In summary, we could speculate that FAK upregulates the expression level of RhoA-GTP in TNF-α-evoked signaling events that gave rise to Ezrin protein phosphorylation in PMVECs.

## Discussion

Up to now, the study of pulmonary vascular ECs focuses mainly on pulmonary macrovascular ECs, and few PMVECs are used as the research subjects. In fact, pulmonary macro- and microvascular ECs exhibit unique structural and functional attributes, even under similar environmental conditions (King et al., 2004). Moreover, PMVECs have more stringent barrier restriction than macrovascular ECs, such as lower protein and water flux and higher TER (Parker and Yoshikawa, 2002). Beck et al. exhibited that PMVECs are more susceptible to inflammatory stimulation and produce more inflammatory chemokines than umbilical vein ECs (Beck et al., 1999). The adhesion rate of polymorphonuclear granulocytes to PMVECs is higher than that of umbilical vein ECs in venous blood flow induced by TNF-α (Otto et al., 2001). That is to say, PMVECs are the main target of inflammatory immune mediators. So, it is more important to explore the inflammatory immune response to PMVECs than to pulmonary macrovascular ECs for clarifying the potential mechanism of ARDS. In this study, we systematically investigated the role of Ezrin protein on endothelial permeability in TNF-α-stimulated PMVECs. Moreover, FAK, RhoA, or their combination was considered as the required signal mechanism, which differed from the previous works (Koss et al., 2006; Shao et al., 2013; Fei et al., 2019; Tang et al., 2021). The process can be demonstrated as follows.

Firstly, it was reported that under the condition of systemic inflammatory reaction, the concentration of plasma TNF-α in patients ranged from 10 to 1,510 pg/ml, which is much lower than that *in vitro* experiments (Wong et al., 1999). Hence, a low concentration of TNF-α (20 ng/ml) was adopted in this study. As a result, a similar trend to that in our previous observations stimulated by 100 ng/ml TNF-α was observed (Tang et al., 2021).

The phosphorylated Ezrin protein was localized primarily along the PMVECs periphery, indicating that the molecule might be involved in the regulation of TNF-α-induced endothelial response, such as cytoskeletal and permeable change. In order to determine the authenticity of this hypothesis, we detected the effect of Ezrin protein by inhibiting its expression using shRNA. The data demonstrated that when the expression level of TNF-α-induced Ezrin protein phosphorylation was elevated, both the cytoplasmic F-actin and cell permeability increased subsequently. On the contrary, the inhibition of Ezrin protein *via* shRNA led to a decrease in phosphorylated Ezrin protein, F-actin, and cell permeability. These phenomena supported the importance of the phosphorylated Ezrin protein in hyperpermeability after TNF-α treatment.

In order to verify the role of phosphorylated Ezrin protein on TNF-α-induced endothelial response, Ezrin^T567A^ was applied. The data showed that F-actin and permeability were significantly inhibited in Ezrin^T567A^ plus TNF-α group compared with Ezrin^WT^ plus TNF-α group, supporting that phosphorylated Ezrin protein had a direct regulatory effect on hyperpermeability in PMVECs. However, the mechanisms underlying Ezrin protein-mediated cytoskeleton reorganization and permeability enhancement remain to be determined. We speculated the following two cases: 1) Ezrin protein directly promotes *de novo* F-actin polymerization, similar to that of phagosomes (Defacque et al., 2000); 2) Ezrin protein plus Rho regulator Rho GDP-dissociation inhibitor (GDI) trigger the intermediate signaling events, such as Rho GTPases, to alter the cytoskeleton and increase the permeability (Takahashi et al., 1997). This theory would be investigated in the future.

Secondly, studies have shown that the activation of RhoA promotes the endothelial hyperpermeability (Spindler et al., 2010; Adderley et al., 2015). Intriguingly, there have been some different views. For instance, Szulcek et al. showed that the activation of RhoA in the periphery of the cell is related to the integrity of the barrier and contributes to the destruction of the barrier in the perinuclear region of the ECs (Szulcek et al., 2013). Moreover, S1P increased the expression level of RhoA-GTP (Breslin et al., 2015), which enhanced the barrier of human umbilical vein ECs and human skin microvascular ECs (Zhang et al., 2016). In the case of the FAK signaling pathway, whether FAK destroys or protects the endothelial barrier is yet controversial (Holinstat et al., 2006; Jean et al., 2014; Alexopoulou et al., 2017). Therefore, the roles of RhoA and FAK signaling pathways in barrier destruction are controversial.

Through the application of inhibitor C3 transferase, we observed that RhoA can enhance the expression level of phosphorylated Ezrin protein and permeability in PMVECs, considering that RhoA may be the upstream signal of phosphorylated Ezrin protein. Actually, not only RhoA but also RhoB and C can be inhibited by C3 transferase. Although RhoA, B, and C are highly homologous, some scholars believed that they play different regulatory roles in endothelial permeability (Kroon et al., 2013; Marcos-Ramiro et al., 2016; Pronk et al., 2019), so to demonstrate the role of RhoA more strictly, we further used RhoA shRNA. It was also found that TNF-α-activated RhoA could upregulate the expression level of phosphorylated Ezrin protein, followed by the remodeling of cytoskeleton and the increased endothelial permeability in PMVECs. The similar analysis of FAK can be done using FAK inhibitor and FAK shRNA. Our examination displayed that the activation of FAK initiated by TNF-α can strengthen the F-actin and the permeability of PMVECs. Accumulating evidence showed that TNF-α-induced phosphorylation of Ezrin protein requires RhoA and FAK signaling pathways; both RhoA and FAK are involved in barrier destruction in PMVECs. The different roles of RhoA and FAK in the endothelial barrier are speculative due to different stimulating factors and endothelia (Bogatcheva et al., 2011).

Finally, we speculated whether the two signaling pathways existed alone or in a combination in TNF-α-challenged PMVECs and which one lies upstream in the latter case? It was found that RhoA shRNA did not inhibit FAK, but the activity of RhoA was attenuated by FAK shRNA, suggesting that the phosphorylation of Ezrin protein in response to TNF-α occurred in a FAK/RhoA-dependent manner.

In addition, the present study has some limitations: 1) how do Ezrin protein cause cytoskeleton reorganization and permeability enhancement has not yet been analyzed in-depth, which is also one of our interests for future research. 2) The PMVECs utilized in this study were cultured *in vitro*, which might not mimic the actual pulmonary microvascular endothelial barrier *in vivo*. However, given the critical role of Ezrin protein in stabilizing the endothelial barrier, altering Ezrin protein and its related signals would be a desirable therapeutic approach.

## Conclusion

In conclusion, we displayed the TNF-α-activated Ezrin protein in rat PMVECs. Phosphorylated Ezrin protein is required for the TNF-α-initiated change in the cytoskeleton and permeability, as well as the downstream targets of FAK/RhoA signaling events in the FAK/RhoA-dependent manner.

## Data Availability

The original contributions presented in the study are included in the article/Supplementary Material; further inquiries can be directed to the corresponding author.
