# Supplementary figures and images for: Inflammatory Immune Cytokine TNF-α Modulates Ezrin Protein Activation via FAK/RhoA Signaling Pathway in PMVECs Hyperpermeability
Source: Front Pharmacol. 2021 May 12;12:676817. doi: 10.3389/fphar.2021.676817 (PMC8152434; doi:10.3389/fphar.2021.676817)

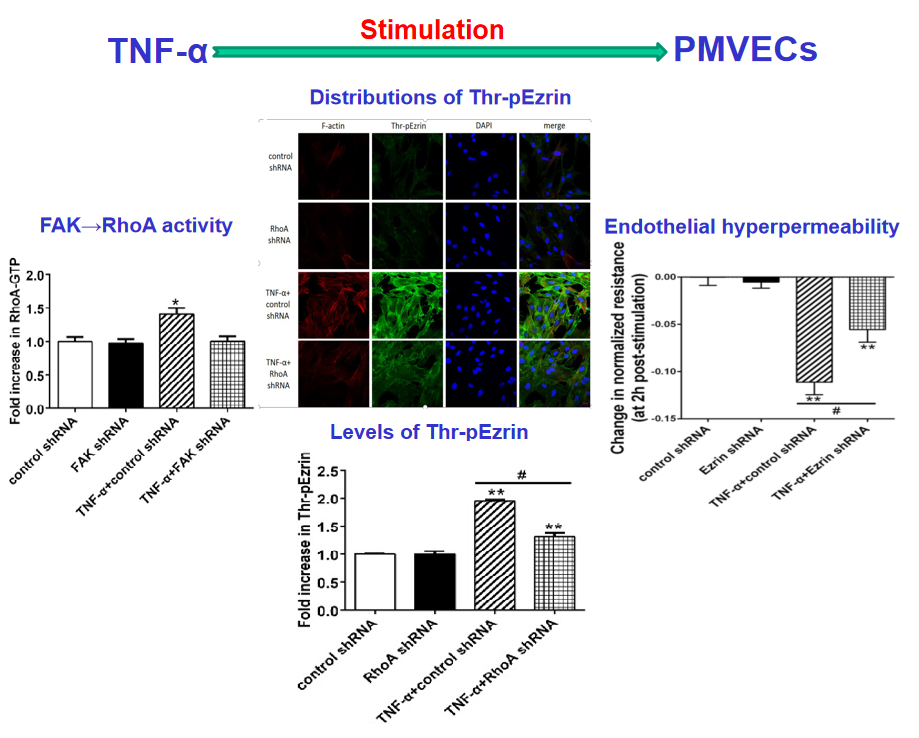

Supplement: Supplementary file 1 [file image1.tif]
